# Bidirectional coupling of physiology and emotions reveals unique speaker–listener dynamics

**DOI:** 10.1093/scan/nsag019

**Published:** 2026-05-06

**Authors:** Mohanad Alkhodari, Ioannis Ziogas, Jace Singh, Paolo Grigolini, Glenn Muschert, Fedor Kusmartsev, Herbert F Jelinek

**Affiliations:** Department of Medical Sciences, Khalifa University, Abu Dhabi, 127788, United Arab Emirates; Cardiovascular Clinical Research Facility (CCRF), Radcliffe Department of Medicine, University of Oxford, Oxford, OX3 9DU, United Kingdom; Healthcare Engineering Innovation Group (HEIG), Khalifa University, Abu Dhabi, 127788, United Arab Emirates; Center for Nonlinear Science, University of North Texas, Texas, 76203, United States; Center for Nonlinear Science, University of North Texas, Texas, 76203, United States; Healthcare Engineering Innovation Group (HEIG), Khalifa University, Abu Dhabi, 127788, United Arab Emirates; Department of Public Health and Epidemiology, Khalifa University, Abu Dhabi, 127788, United Arab Emirates; Department of Physics, Khalifa University, Abu Dhabi, 127788, United Arab Emirates; Department of Medical Sciences, Khalifa University, Abu Dhabi, 127788, United Arab Emirates; Healthcare Engineering Innovation Group (HEIG), Khalifa University, Abu Dhabi, 127788, United Arab Emirates

**Keywords:** bidirectional coupling, physiological synchronization, emotional dynamics, heart rate variability, entropy and complexity

## Abstract

Conversational interactions extend beyond verbal exchange, involving dynamic synchronization across physiological, emotional, and acoustic domains. This study aimed to characterize multimodal speaker-listener coupling during structured debates to examine how bidirectional dynamics relate to autonomic regulation, signal complexity, and emotional states. We analyzed data from the K-EmoCon database, which included 32 participants in 10-minute debates. Heart rate variability (HRV) features, speech features, and multi-perspective emotion annotations were synchronized and analyzed in segmented speak-listen phases. Cross-correlation and bidirectional coupling quantified coupling strength and directionality, and their relationships with emotional states were assessed. Negative lag segments showed significantly higher HRV features, including AVNN (0.655 [0.631–0.749], *P *= .003), HF power (0.620 [0.338–0.781], *P *< .001), and SD1 (1.30 × 10^−3^ [1.19–1.41] × 10^−3^, *P *= .003). Positive lag segments were associated with higher sample entropy (0.058 [0.046–0.065], *P* < .001) and diffusion entropy (μ_r_: 1.209 [1.201–1.221], *P* < .001). Lower emotion states (1–2) tended to exhibit negative-lag dynamics, whereas moderate to high states (3 and 5) showed a modest positive-lag predominance. Multimodal coupling reveals distinct physiological and emotional signatures linked to leadership and responsiveness in conversation, providing insights for therapeutic, educational, and collaborative applications.

## Introduction

Conversational interactions extend beyond being only verbal communications; they also involve complex and dynamic synchronization between partners across multiple physiological and neural systems, including cardiovascular, respiratory, and brain activities (Schoot *et al.* 2016). In conversations, the exchange of emotions and physiological responses between speakers and listeners reflects the level of alignment or coupling between interlocutors (Lin *et al.* 2024). Usually, stronger coupling indicates stronger interaction, while weaker coupling signals misalignment or breakdowns in interaction between participants (Li *et al.* 2021). Speaker–listener coupling is increasingly recognized as a core mechanism underlying effective communication in settings such as discussions, debates, and collaborative meetings (Jiang *et al.* 2021).

Inter-individual coupling mechanisms extend beyond physiological synchronization but also incorporate affective and psychological domains. Emotional states can be transmitted between a speaker and a listener, which can influence how information is received and interpreted (Alhussein *et al.* 2025). Consequently, recognizing emotions in dialogic contexts is essential for capturing the interaction of cognitive function associated with speaker–listener paradigms and affect during communication. Coupling has implications for the design of empathetic algorithms and computational models aimed at predicting conversational outcomes (Peräkylä *et al.* 2015). Research in this area has approached the problem from diverse perspectives, including semantic analyses of speech content (Dikker *et al.* 2014), acoustic and prosodic cues (Eger and Reinisch 2019), and physiological signals such as respiratory patterns and cardiovascular dynamics (Orepic *et al.* 2022, Divjak *et al.* 2024). However, further characterization is needed through multimodal approaches that integrate speech and physiology during both speaking and listening phases to better understand the inherent complexity of interpersonal alignment.

Physiological signals provide information about the psychological processes that accompany social interaction, including electrocardiography (ECG) and derived heart rate variability (HRV), which have been applied to study stress, attention, and affective states during conversation (Wang *et al.* 2023). HRV reflects the balance between the sympathetic and parasympathetic autonomic nervous system activity and is a well-established marker of emotional regulation and adaptability (Alkhodari *et al.* 2024). Besides ECG and HRV, photoplethysmography (PPG) analysis is a non-invasive method for assessing cardiovascular dynamics (Armañac-Julián *et al.* 2025), which provide reliable estimates of HRV during conversations. Electroencephalography (EEG) has also been employed to capture brain-to-brain coupling between interlocutors (Li *et al.* 2023).

Entropy-based measures of speech have been linked to inter-brain dynamics and have been investigated to describe how neural mechanisms facilitate alignment in dialogue (Hull and Bruce Morton 2023). By quantifying the complexity and unpredictability of speech patterns, entropy provides an indirect measure of the underlying cognitive and emotional states. Applying diffusion entropy analysis to characterize the diffusion process also provides insight into crucial events (Shah 2024), which are defined as short intervals of turbulence that would otherwise remain undetected. These events reflect the adaptability of functional outcomes in both speech and physiological processes(Culbreth *et al.* 2019). Speech that contains crucial events exhibits a higher diffusion scaling parameter than speech without such events. The scaling parameter derived from the statistical analysis of crucial events, therefore, serves as an indicator of the adaptive capacity of verbal output or physiological signalling (Li *et al.* 2023, Armañac-Julián *et al.* 2025). Despite these advances, limited information is available on how interpersonal variability in speaker–listener speech patterns can be characterized, particularly in contexts of emotional, physiological, and psychological interactions.

**Figure 1:**
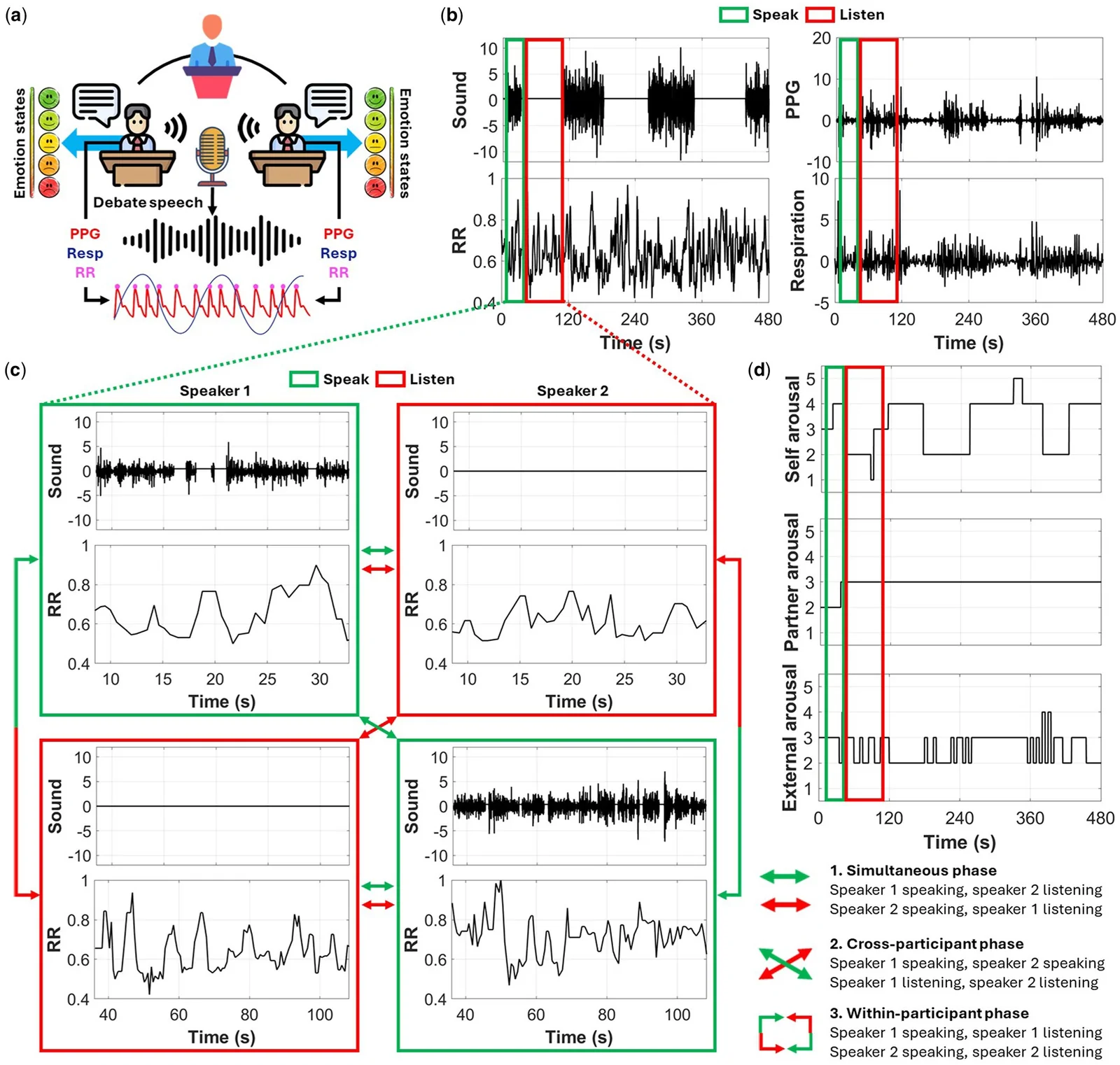
Overview of the interpersonal coupling analysis pipeline. (a) An illustration of the conversational environment of the K-EmoCon dataset and the type of data acquired. (b) Example taken from the dataset (group 9, participant 1) showing speech and the corresponding physiological signals. The signals were divided into speaking (green) and listening (red) phases. (c) Zoomed-in demonstration of the speaking-listening analysis, including three major phases, namely 1) simultaneous, cross-participant, and within-participant phases. (d) The corresponding emotional arousal states from self, partner, and external perspectives.

In this study, we investigated multimodal speaker–listener coupling during structured debates by integrating physiological, speech, and emotion data (Fig. 1). Instead of analyzing entire debate recordings and their corresponding emotional and physiological signals, the conversations were divided into distinct phases based on speaking and listening segments between two participants. This phase-based approach allows us to characterize how bidirectional coupling relates to autonomic regulation, signal complexity, and emotional states, rather than focusing on isolated feature-level effects. Accordingly, we utilized a theoretically motivated set of HRV features, entropy dynamics, and speech characteristics to capture complementary aspects of multimodal synchrony across interaction phases.

## Methods

### Dataset and study cohort

The dataset used in this study was drawn from the publicly available K-EmoCon (Park *et al.* 2020) multimodal database, which consists of English-language debates on the socially sensitive issue of accepting refugees from Yemen in Korea. Thirty-two participants aged 19 to 36 were randomly assigned to 16 dyads, each engaging in a debate session lasting approximately 10 minutes. To capture the complexity of human emotional expression, the dataset includes PPG, along with audio recordings of speech and video recordings of facial expressions and body movements (Alhussein *et al.* 2025).

Emotion annotations were systematically collected at 5-second intervals from three complementary perspectives: (i) self-reports provided by the speaker reflecting their own perceived emotional state, (ii) partner-reports in which the listener assessed the speaker’s emotions during the interaction, and (iii) external evaluations contributed by five independent raters who observed the recorded sessions. These multi-perspective annotations were projected onto the continuous affective dimensions of arousal and valence, indicative of changes in the emotional dynamics during the debate. Both arousal and valence were quantified on a five-point scale (1–5). Arousal reflects the level of emotional activation or intensity, ranging from low (1—calm) to high (5—excited), whereas valence represents the emotional polarity of the experience, ranging from negative (1—low values) to positive (5—high values).

### Physiological signals preparation and pre-processing

#### PPG recordings

PPG were obtained using the Empatica E4 wristband at a sampling rate of 64 Hz. The recordings were first cropped to the start and end times defined in the metadata to ensure consistency across subjects. To improve signal quality and reduce noise, the raw PPG was processed with a 4th-order zero-phase Butterworth band-pass filter (0.5–5 Hz), which preserved the frequency band relevant to pulse rhythm while attenuating motion artifacts and baseline drift (Lapitan *et al.* 2024).

#### Interbeat interval sequences

Peak detection was then performed on the filtered PPG to extract beat-to-beat intervals (here denoted as RR intervals) from successive peaks. Peaks were identified using the MATLAB “*findpeaks*” function with a minimum peak distance of 0.4 s, corresponding to a maximum physiologically plausible heart rate of 150 bpm. This threshold is conservative and commonly adopted in HRV analysis for seated, non-exertional tasks to minimize artifacts in PPG signals (Sacha and Pluta 2008). To reduce noise and correct for spurious detections, intervals were pre-processed using the SDROM algorithm following the approach described in our previous work (Saleem *et al.* 2022). The cleaned data was visually inspected to confirm signal quality and physiological plausibility, with the vast majority of instantaneous heart rate values falling below 150 bpm. The resulting RR series (normal-to-normal [NN] peaks) was then interpolated to the continuous PPG timeline, producing frame-level RR values synchronized with the original recordings. Furthermore, heart rate (HR) was estimated continuously and used to adjust the extracted features by fitting a linear regression model.

#### Respiratory signal

Respiration was estimated from the PPG using the approach described by Charlton *et al.* (2017) Accordingly, we derived the following: baseline wander, reflecting low-frequency shifts in the PPG baseline caused by thoracic movements; amplitude modulation, quantified with the Hilbert envelope to capture respiration-induced variations in pulse amplitude; and frequency modulation, representing respiratory sinus arrhythmia observed in beat-to-beat interval fluctuations. Each component was band-pass filtered within the expected respiratory frequency range of 0.1–0.7 Hz (Leiva *et al.* 2025), normalized, and then averaged to form a single composite respiration signal. The respiratory signal was employed to estimate the respiratory rate, and applying a linear regression model to remove respiration-related artifacts.

### Speech recordings and speak-listen segmentation

To incorporate speech activity and physiological data, debate audio streams were pre-processed (Ziogas *et al.* 2024). Briefly, because K-EmoCon was collected in natural conversational settings, the raw audio contained overlapping speech, referee interventions, and non-conversational noise. To address this, a source-separation–focused preprocessing strategy was adopted. Recordings were first max-scaled, and silent segments were removed using short-time Fourier transform (STFT) energy thresholding. Segments containing speech were then manually annotated and processed with either the WTST-NST filter applied to suppress stationary noise (Hadjileontiadis *et al.* 2000), or a pre-trained Sepformer model (trained on Librimix) for overlapping voices, depending on the noise profile (Subakan *et al.* 2023).

Building on this framework, the debate audio was prepared for integration with the physiological signals in the analysis. Extracted segment annotations were used to divide each debate into speaker-specific tracks, which were subsequently mapped from the original 22.5 kHz sampling rate to the 64 Hz timeline of the physiological recordings such as PPG, RR, and respiration. From this aligned representation, two sets of features were derived: (i) binary speaking indicators for both participants, denoting whether each participant was actively speaking within a given frame, and (ii) continuous intensity traces for both participants, capturing the relative amplitude of the vocal activity of each speaker. This synchronization step provided speech features at the debate level, aligned with physiological and emotional annotations.

### Alignment of emotion annotations

To synchronize the discrete emotion annotations in K-EmoCon with the continuously recorded physiological signals, each 5-second label was expanded across its corresponding interval and repeated over all frames within that window. Annotation timestamps (in seconds) were directly matched to the relative time of the PPG recordings, ensuring precise temporal alignment. This up-sampling process provided frame-level emotion sequences for arousal and valence, aligned to the 64 Hz sampling rate of the PPG data. For the external evaluation, we used the aggregated ratings originally provided in the dataset (“aggregated_external_annotations”), obtained by combining decisions of five independent annotators using a majority-voting procedure to produce a single consensus label per instance (Park *et al.* 2020).

### Feature extraction

**Figure 2:**
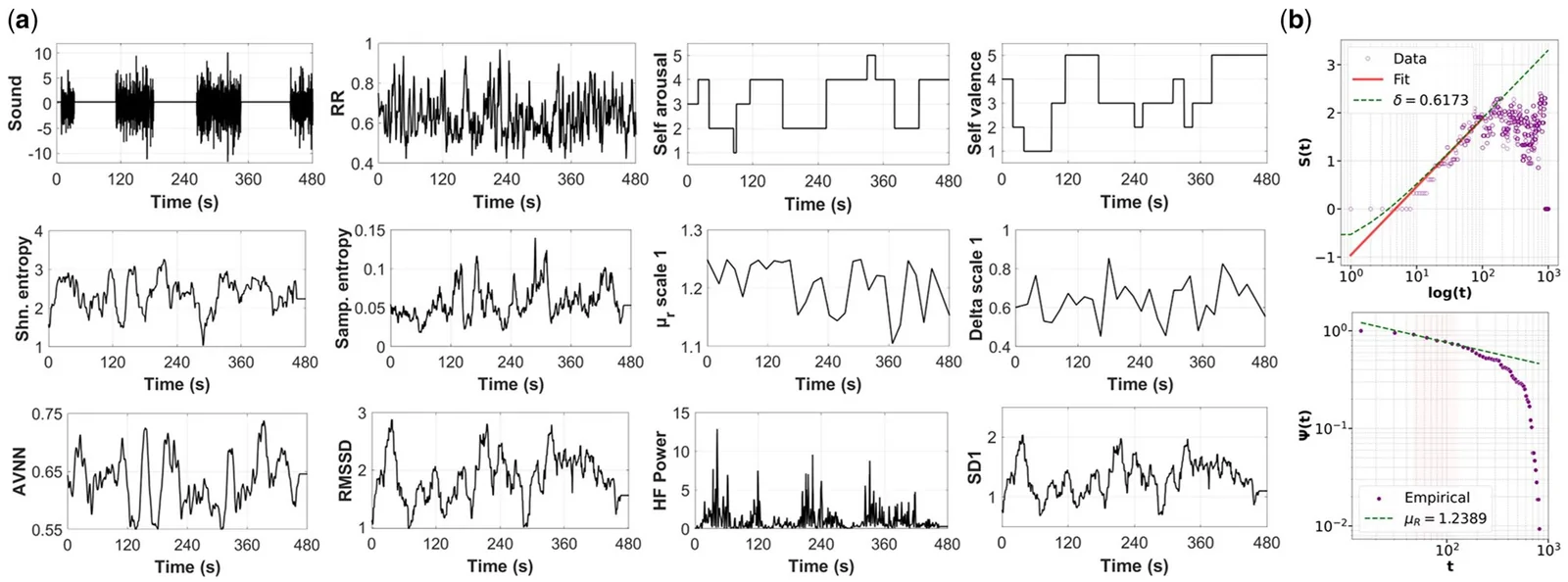
Example features extracted from the peak-to-peak (RR) intervals. (a) Feature extracted from the RR data shows synchrony with the emotional states (self, arousal, and valence), including Shannon entropy, sample entropy, diffusion entropy, and heart rate variability (HRV). (b) Segment results from the diffusion entropy analysis showing the scaling exponent (delta, δ) and the complexity index (μ_r_).

Information-theoretic descriptors were derived from PPG, RR, and speech to quantify signal complexity and conduct comparative analysis. Features were computed sequentially within sliding windows of 1000 samples, advanced with a one-sample at a time, resulting in a continuous feature vector temporally aligned with the original recordings. The types of features extracted are briefly outlined below and demonstrated with examples in Fig. 2.

### Analysis of entropy

We calculated Shannon entropy as a measure of the average information content in the probability distribution of the signal, reflecting its overall unpredictability (Feutrill and Roughan 2021). Sample entropy, which quantifies time-series irregularity by measuring the likelihood that similar sequences remain similar when extended, was computed with *m* = 2m and *r* = 0.2 (Patel *et al.* 2021). Rényi entropy was included to generalize the Shannon formulation by varying the parameter “α”, which tunes the sensitivity to events of different probabilities (Cornforth *et al.* 2013). Specifically, Rényi entropy was calculated for a range of scales between −5 and +5 (α ∈ {−5, −3, −1, 1, 3, 5}), where negative values emphasize contributions from rare events, positive values emphasize frequent events, and α = 1 approximates Shannon entropy. All entropies were computed using a global histogram with 10 bins per recording to maintain comparability across windows.

In addition, we applied multiscale diffusion entropy analysis (MSDEA) as previously described (Nasrat *et al.* 2023) to characterize the dynamical properties of the signals. For MSDEA, the series were coarse-grained at monotonically increasing dyadic scales (4, 8, 16, 32, and 128) using block averaging, and diffusion entropy was estimated for each coarse-grained representation. From this analysis, two diffusion entropy-based descriptors were derived in accordance with the definitions of Grigolini et al. (Jelinek *et al.* 2020, Benfatto *et al.* 2021). First, the scaling exponent (delta, *δ*), which characterizes entropy growth across diffusion times, was derived. Moreover, the complexity index (*μ_r_*), indicating the presence of crucial events, can be derived from *δ*, and reflects the degree of dynamical complexity and crucial events in the signal.

Here, *δ* and *μ_r_* are linked by the following equations:


$$\delta =\frac{1}{1-\mu_{r}},\;\mu_{r}=\;1+\frac{1}{\delta }$$

It attains the maximum value of *δ* = 1 for *μ_r_* ≥ 2, interpreted as a manifestation of maximum intelligence (adaptability) and as a crucial event in the time series.

#### Heart rate variability

We computed a standard set of HRV features from the RR series (Alkhodari *et al.* 2021). Time-domain measures included the mean of RR intervals (AVNN), their standard deviation (SDNN), the root mean square of successive differences (RMSSD), the percentage of intervals differing by more than 50 ms (pNN50), and the standard error of the mean (SEM). Frequency-domain features were obtained from the power spectral density estimated via Welch’s method (Krafty *et al.* 2014) with overlapping windows, comprising absolute and normalized powers in the very-low-frequency (VLF), low-frequency (LF), and high-frequency (HF) bands, peak frequencies within each band, the LF/HF ratio, and total power. Nonlinear indices were derived from the Poincaré plot (SD1 and SD2) and detrended fluctuation analysis (DFA) was applied to obtain a short (α1) and long (α2) range correlation indices (Zhang *et al.* 2019).

#### Speech pitch features

To capture speech-related acoustic dynamics, we extracted pitch from the debate audio streams on a per-speaker basis. The pitch was computed using the normalized cross-correlation function (NCF) implemented in MATLAB’s *pitch* function, with a 40-ms analysis window and a search range of 50-300 Hz. The resulting pitch signal was initially estimated for the full audio to ensure robust voicing detection. Then, it was aligned to the standard 64 Hz timeline of the physiological and emotional annotation streams. We aligned pitch to speech signals using cross-correlation within a ± 1-second window to correct any temporal mismatches introduced by window-based pitch detection. Finally, speaker-specific pitch signals were obtained by gating the overall extracted pitch with each participant’s speaking mask, resulting in two synchronized pitch features (*pitch_1*, *pitch_2*).

From the speaker-specific pitch signals, we extracted features within sliding windows of 1000 samples (Alhussein *et al.* 2025). For each subject, the corresponding pitch stream was analyzed to obtain the mean and standard deviation as measures of central tendency and variability, and skewness and kurtosis to capture distributional asymmetry. Additionally, Shannon entropy was computed from the pitch distribution within each window, providing an estimate of the unpredictability of pitch fluctuations.

### Cross-correlation analysis

To investigate interpersonal coupling, we carried out cross-correlation analysis of participants’ RR sequences and speech signals within debate segments defined by speaking and listening activity. Each segment was extracted based on the binary speaking indicators and analyzed separately to preserve the temporal structure of the interactions. We examined coupling across three complementary perspectives. First, we assessed simultaneous synchrony, in while one participant spoke and the other listened. Second, we analyzed cross-participant interactions when both participants were in the same state, either both speaking or listening, to capture potential parallel coordination. Finally, we examined within-participant dynamics by comparing each individual’s RR patterns during their own speaking versus listening intervals.

To measure the connection between participants, we applied unbiased cross-correlation analysis to RR and speech signals and the method described by Reynolds and Osler (2014), in which signals were first detrended to remove non-stationary drift and then normalized, ensuring comparability across participants. Cross-correlation was then calculated by systematically shifting one participant’s time series relative to the other and computing the Pearson correlation coefficient at each temporal offset. The analysis ensured that the autocorrelation at zero lag equalled one, allowing direct interpretation of the coupling strength between −1 and 1. From each function, we extracted two key descriptors: the corresponding lag, reflecting temporal directionality, and the maximum correlation coefficient, reflecting the strength of interaction. Specifically, a positive lag indicates that the signal of the first participant preceded that of the second, whereas a negative lag indicates that the first participant’s signal followed the second participant’s signal. Positive correlation values indicate that both signals tend to vary in the same direction over time, i.e., increases and decreases occur together), whereas negative correlation values indicate inverse coupling, where increases in one participant’s signal are associated with decreases in the other.

Analyses were performed separately for each debate segment defined by speaking and listening states, which allowed us to capture both across-participant coordination and within-participant modulation between speaking and listening. In addition, overlapping speaking periods were analyzed using the same procedure to assess acoustic synchrony.

### Statistical analysis

We performed statistical analysis to evaluate group differences and relationships between features from different perspectives. A Kruskal–Wallis test was applied to assess whether there were significant differences across groups without assuming normality, making it suitable for non-parametric data (McKight and Najab 2010). All *P*-values were adjusted using the Bonferroni method to control the family-wise error rate. The significance of the Pearson correlation was assessed using a *t*-test on the correlation coefficient, with the resulting *P*-value indicating whether the observed association was statistically meaningful (Komaroff 2020). Significance was defined as a *P*-value below .05; however, we also allowed the threshold to go below 0.1 for further interpretations.

## Results

### Analysis of cross-correlation

**Figure 3:**
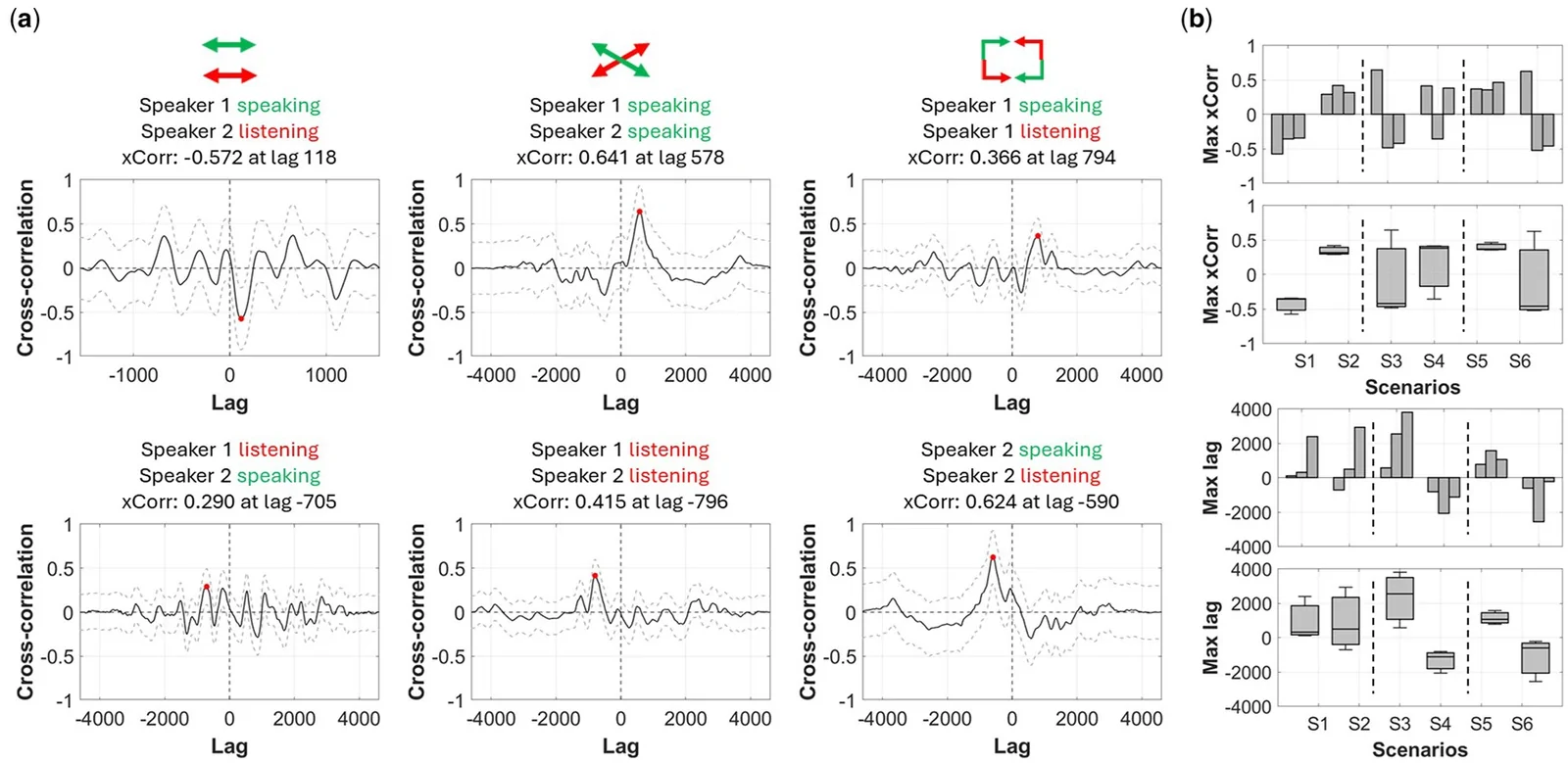
Example results from the cross-correlation (xCorr) analysis. (a) The cross-correlation analysis between peak-to-peak (RR) sequences for the three phases is demonstrated on each column for the first speaking-listening segment. (b) The cumulative results of the same example for the three speaking-listening segments of the conversation for each phase. The results show bars corresponding to the value of cross-correlation and lag on each segment alongside the boxplot representation of the combined three segments.

The results of the cross-correlation analysis are illustrated for a single speak-listen segment from one of the debates (Fig. 3a). In this example, the cross-correlations across all three phases showed opposite lag directions and opposite correlation patterns during the simultaneous speak-listen phase (left column). When aggregated across all segments (Fig. 3b), a consistent pattern emerged across the three phases of the debate, characterized by distinct negative or positive lags and corresponding correlations.

#### Lag directionality and physiological dynamics

**Figure 4:**
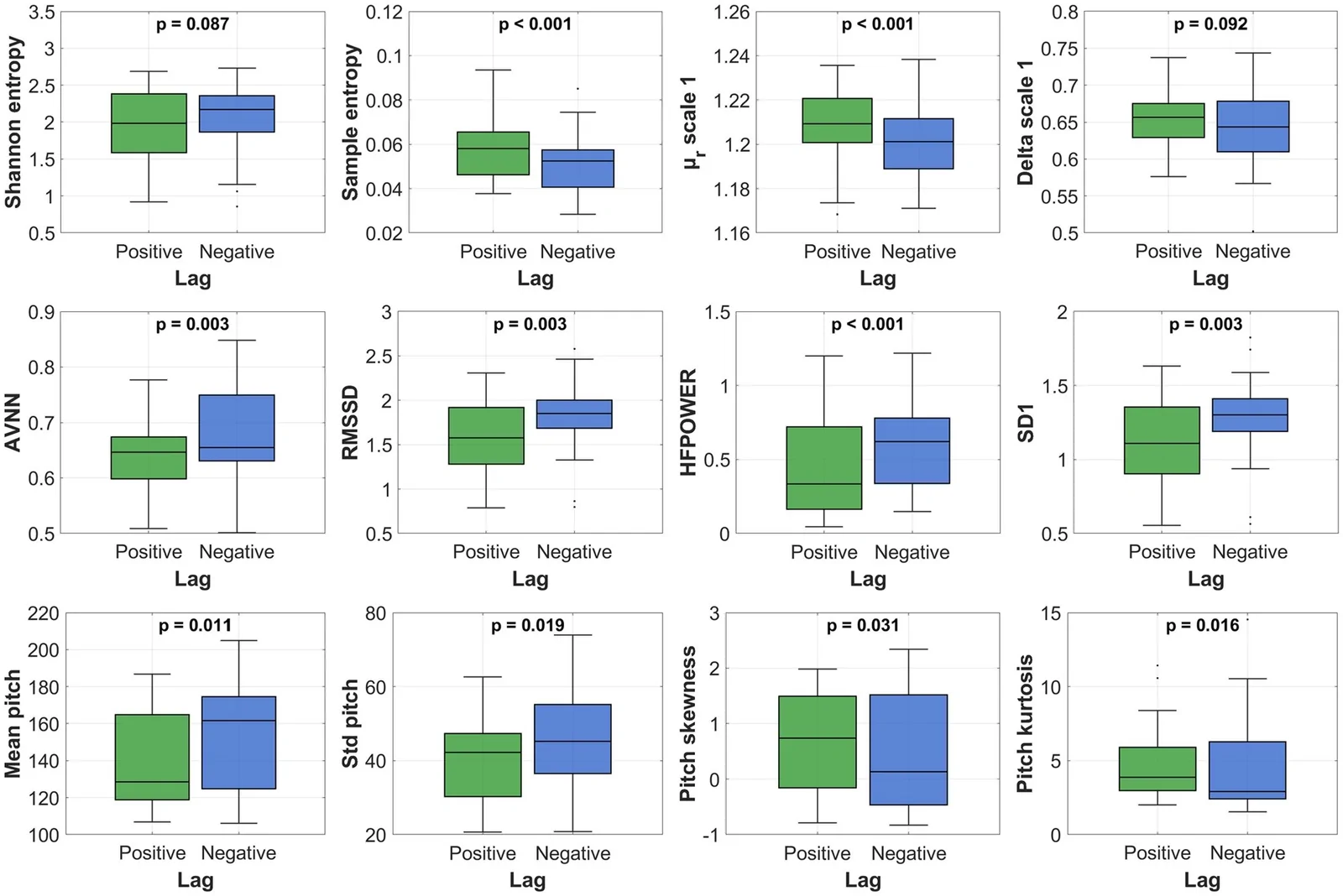
Significantly different features extracted from peak-to-peak (RR) intervals at positive and negative lags across all speaking-listening phases. Significance was tested through the Kruskal–Wallis test. Positive lag indicates that the signal of the first participant preceded that of the second, whereas a negative lag indicates that the first participant’s signal followed the second participant’s signal.

When negative and positive lag segments were aggregated with their corresponding physiological feature values, the summary for each feature revealed distinct dynamics (Fig. 4). For instance, negative lag segments generally exhibited significantly higher HRV characteristics, including higher AVNN (0.655 [0.631–0.749], *P* = .003), RMSSD (1.85 × 10^−3^ [1.69–2.00] × 10^−3^], *P* = .003), HF power (0.620 [0.338–0.781], *P* < .001), and SD1 (1.30 × 10^−3^ [1.19–1.41] × 10^−3^, *P* = .003). They also showed higher Shannon entropy (2.173 [1.863–2.360], *P* = .087) compared to the positive lag segments. In contrast, positive lag segments demonstrated significantly higher sample entropy (0.058 [0.046–0.065], *P* < .001) and diffusion entropy, as indicated by μ_r_ (1.209 [1.201–1.221], *P* < .001) and δ (0.656 [0.629–0.675], *P* = .092). Notably, negative lag segments were also associated with significantly higher mean and standard deviation of pitch (*P* < .02), as well as lower skewness and kurtosis (*P*  < .03). A summary of these features is provided in Table 1.

**Table 1:** Key significantly different features summarized by positive and negative lag or correlation.

Features	Positive lag	Negative lag	*P*-value
Based on lag directionality			
Shannon entropy	1.986 [1.589–2.381]	2.173 [1.863–2.360]	.087
Sample entropy	0.058 [0.046–0.065]	0.052 [0.041–0.058]	<.001
μ_*r*_ scale 1	1.209 [1.201–1.221]	1.201 [1.189–1.212]	<.001
Delta scale 1	0.656 [0.629–0.675]	0.644 [0.610–0.678]	.092
AVNN	0.646 [0.598–0.674]	0.655 [0.631–0.749]	.003
RMSSD	1.57 × 10^−3^ [1.28–1.92] × 10^−3^	1.85 × 10^−3^ [1.69–2.00] × 10^−3^	.003
HFPOWER	0.333 [0.163–0.722]	0.620 [0.338–0.781]	<.001
SD1	1.11 × 10^−3^ [0.90–1.35] ×10^−3^	1.30 × 10^−3^ [1.19–1.41] ×10^−3^	.003
Pitch mean	127.034 [118.462–163.784]	161.641 [124.812–174.597]	.003
Pitch std	38.325 [29.192–46.413]	45.290 [36.563–54.959]	.004
Pitch skewness	0.782 [−0.044 to 1.566]	0.196 [−0.464 to 1.420]	.031
Pitch kurtosis	4.675 [3.120–7.486]	2.918 [2.425–6.273]	.016
Based on correlation directionality			
Shannon entropy	1.993 [1.583–2.439]	2.168 [1.843–2.276]	.975
Sample entropy	0.052 [0.045–0.063]	0.054 [0.042–0.058]	.011
μ_r_ scale 1	1.211 [1.201–1.221]	1.201 [1.190–1.212]	<.001
Delta scale 1	0.642 [0.625–0.670]	0.644 [0.611–0.678]	.412
AVNN	0.660 [0.636–0.675]	0.653 [0.625–0.721]	.378
RMSSD	1.74 × 10^−3^ [1.28–1.99] × 10^−3^	1.81 × 10^−3^ [1.52–2.00] × 10^−3^	.072
HFPOWER	0.454 [0.163–0.725]	0.536 [0.281–0.736]	.040
SD1	1.23 × 10^−3^ [0.90–1.40] × 10^−3^	1.27 × 10^−3^ [0.11–1.41] × 10^−3^	.075
Pitch mean	128.402 [120.281–165.080]	149.686 [122.106–175.929]	.120
Pitch std	42.268 [28.842–46.413]	45.484 [27.865–54.959]	.076
Pitch skewness	0.740 [−0.191 to 1.566]	0.224 [−0.466 to 1.543]	.029
Pitch kurtosis	3.831 [3.119–6.142]	2.934 [2.522–6.273]	.061

#### Lag directionality and emotional states

**Figure 5:**
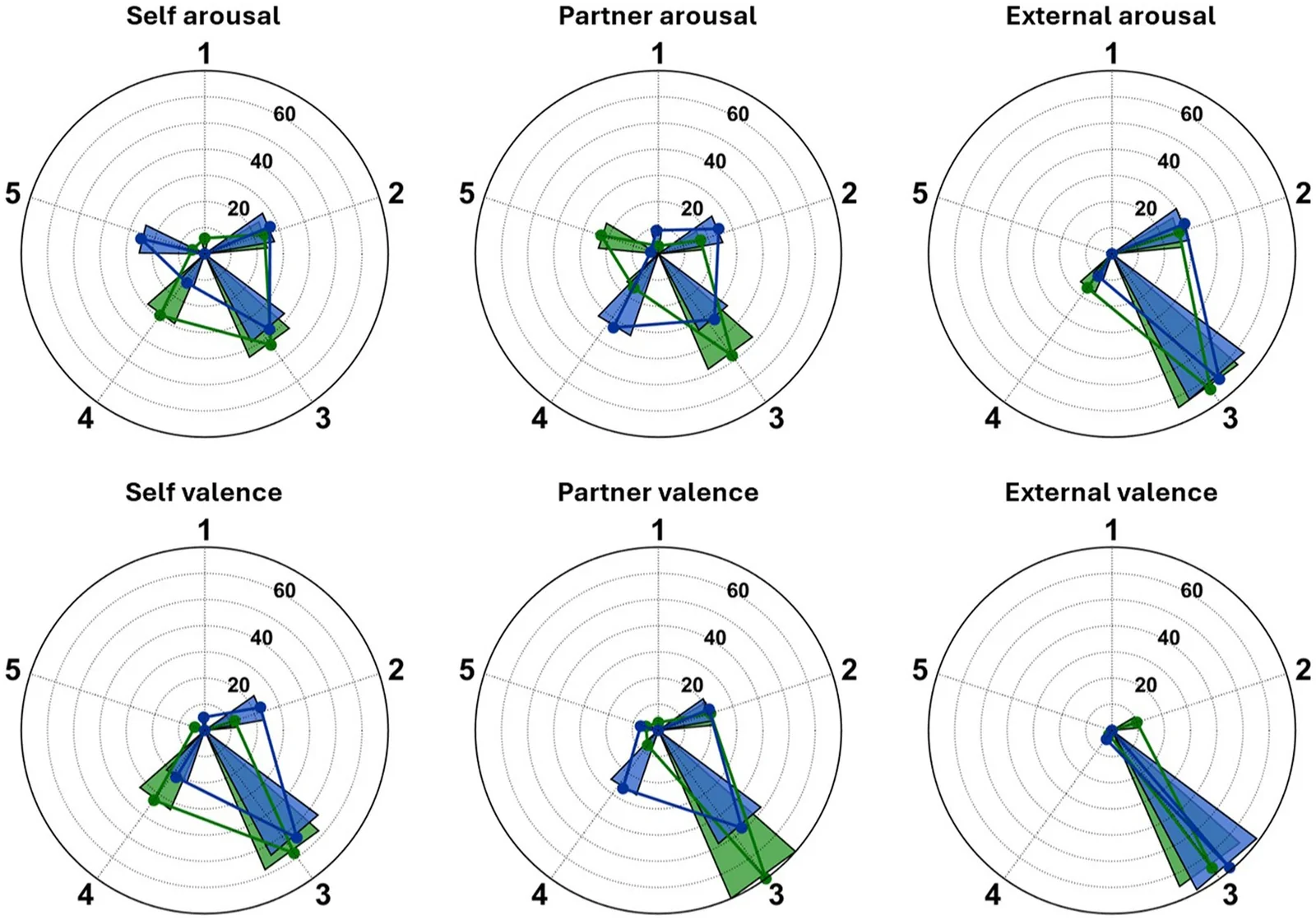
Distribution of positive and negative segments defined by lag (leading or lagging) relative to emotional states in arousal and valence on a scale from 1 to 5. Segments were defined based on speaking-listening phases and matched with their corresponding dominant emotional state. Positive lag indicates that the signal of the first participant preceded that of the second, whereas a negative lag indicates that the first participant’s signal followed the second participant’s signal. Arousal reflects the level of emotional activation or intensity, ranging from low (1—calm) to high (5—excited), whereas valence represents the emotional polarity of the experience, ranging from negative (1—low values) to positive (5—high values)

Emotional states varied for negative and positive lag segments (Fig. 5). Self-reported arousal and valence were characterized by a higher proportion of positive segments in emotional state 4 (*P* < .001). However, an opposite trend was observed for state 5, which reflects greater emotional extremes. For example, arousal exhibited more negative segments in state 5 (*P* < .001), whereas valence showed the reverse pattern (*P* = .029). For partner-reported arousal and valence, state 3 followed a similar pattern to an even greater extent, with more positive segments. In contrast, state 4 showed the opposite trend, being associated with more negative segments (*P* < .001). Notably, the extreme emotional state 5 was again reversed, with a higher proportion of positive segments compared to self-reported (*P* < .001). It is also worth noting that external arousal and valence did not display clear variations across lag directionality. Most observations were concentrated in state 3, which represents the average emotional state. Overall, negative-lag patterns were more common during lower emotional states (1–2), whereas a relative dominance of positive-lag dynamics characterized moderate and high states (3 and 5). A summary of emotion state distribution is provided in Table 2.

**Table 2:** Distribution of emotion segments based on positive and negative lag and correlation directionality.

Emotions	Perspectives	Emotion states				
		1 (Low)	2	3	4	5 (High)
Based on lag directionality						
Arousal	Self	POS: 6 (100.0%)	POS: 24 (47.1%)	POS: 43 (53.1%)	POS: 29 (69.0%)	POS: 5 (16.7%)
		NEG: 0 (0.0%)	NEG: 27 (52.9%)	NEG: 38 (46.9%)	NEG: 13 (31.0%)	NEG: 25 (83.3%)
		***P* = .002**	*P* = .552	*P* = .432	***P* < .001**	***P* < .001**
	Partner	POS: 3 (25.0%)	POS: 17 (40.5%)	POS: 48 (59.3%)	POS: 16 (32.7%)	POS: 23 (88.5%)
		NEG: 9 (75.0%)	NEG: 25 (59.5%)	NEG: 33 (40.7%)	NEG: 33 (67.3%)	NEG: 3 (11.5%)
		***P* = .014**	*P* = .081	***P* = .018**	***P* = .001**	***P* < .001**
	External	POS: 0 (NA)	POS: 27 (47.4%)	POS: 64 (50.4%)	POS: 16 (61.5%)	POS: 0 (NA)
		NEG: 0 (NA)	NEG: 30 (52.6%)	NEG: 63 (49.6%)	NEG: 10 (38.5%)	NEG: 0 (NA)
		*P* = NA	*P* = .574	*P* = .900	*P* = .096	*P* = NA
	Total	POS: 9 (50.0%)	POS: 68 (45.3%)	POS: 155 (53.6%)	POS: 61 (52.1%)	POS: 28 (50.0%)
		NEG: 9 (50.0%)	NEG: 82 (54.7%)	NEG: 134 (46.4%)	NEG: 56 (47.9%)	NEG: 28 (50.0%)
		*P* = 1.000	*P* = .106	*P* = .081	*P* = .513	*P* = 1.000
Valence	Self	POS: 0 (0.0%)	POS: 12 (34.3%)	POS: 58 (51.8%)	POS: 33 (61.1%)	POS: 4 (100.0%)
		NEG: 5 (100.0%)	NEG: 23 (65.7%)	NEG: 54 (48.2%)	NEG: 21 (38.9%)	NEG: 0 (0.0%)
		***P* = .008**	***P = *.009**	*P* = .593	***P* = .021**	***P* = .029**
	Partner	POS: 3 (100.0%)	POS: 21 (50.0%)	POS: 71 (59.2%)	POS: 7 (21.2%)	POS: 5 (41.7%)
		NEG: 0 (0.0%)	NEG: 21 (50.0%)	NEG: 49 (40.8%)	NEG: 26 (78.8%)	NEG: 7 (58.3%)
		*P* = .100	*P* = 1.000	***P *= .005**	***P* < .001**	*P* = .414
	External	POS: 0 (NA)	POS: 10 (100.0%)	POS: 95 (49.0%)	POS: 2 (33.3%)	POS: 0 (NA)
		NEG: 0 (NA)	NEG: 0 (0.0%)	NEG: 99 (51.0%)	NEG: 4 (66.7%)	NEG: 0 (NA)
		*P* = NA	***P* < .001**	*P* = .685	*P* = .567	*P* = NA
	Total	POS: 3 (37.5%)	POS: 43 (49.4%)	POS: 224 (52.6%)	POS: 42 (45.2%)	POS: 9 (56.2%)
		NEG: 5 (62.5%)	NEG: 44 (50.6%)	NEG: 202 (47.4%)	NEG: 51 (54.8%)	NEG: 7 (43.8%)
		*P* = .619	*P* = .879	*P* = .132	*P* = .187	*P* = .480
Based on correlation directionality						
Arousal	Self	POS: 7 (100.0%)	POS: 29 (65.9%)	POS: 55 (59.8%)	POS: 26 (70.3%)	POS: 10 (33.3%)
		NEG: 0 (0.0%)	NEG: 15 (34.1%)	NEG: 37 (40.2%)	NEG: 11 (29.7%)	NEG: 20 (66.7%)
		***P* = .001**	***P* = .003**	***P* = .008**	***P* = .000**	***P* = .010**
	Partner	POS: 2 (22.2%)	POS: 26 (56.5%)	POS: 49 (62.0%)	POS: 21 (45.7%)	POS: 29 (96.7%)
		NEG: 7 (77.8%)	NEG: 20 (43.5%)	NEG: 30 (38.0%)	NEG: 25 (54.3%)	NEG: 1 (3.3%)
		*P* = .057	*P* = .211	***P* = .003**	*P* = .404	***P* < .001**
	External	POS: 0 (NA)	POS: 32 (52.5%)	POS: 74 (61.2%)	POS: 21 (75.0%)	POS: 0 (NA)
		NEG: 0 (NA)	NEG: 29 (47.5%)	NEG: 47 (38.8%)	NEG: 7 (25.0%)	NEG: 0 (NA)
		*P* = NA	*P* = .587	***P* = .001**	***P* < .001**	*P* = NA
	Total	POS: 9 (56.2%)	POS: 87 (57.6%)	POS: 178 (61.0%)	POS: 68 (61.3%)	POS: 39 (65.0%)
		NEG: 7 (43.8%)	NEG: 64 (42.4%)	NEG: 114 (39.0%)	NEG: 43 (38.7%)	NEG: 21 (35.0%)
		*P* = .480	***P* = .008**	***P* < .001**	***P* = .001**	***P* = .001**
Valence	Self	POS: 0 (0.0%)	POS: 18 (43.9%)	POS: 63 (62.4%)	POS: 44 (71.0%)	POS: 2 (100.0%)
		NEG: 4 (100.0%)	NEG: 23 (56.1%)	NEG: 38 (37.6%)	NEG: 18 (29.0%)	NEG: 0 (0.0%)
		***P* = .029**	*P* = .269	***P* < .001**	***P* < .001**	*P* = .333
	Partner	POS: 2 (100.0%)	POS: 28 (75.7%)	POS: 79 (62.7%)	POS: 9 (30.0%)	POS: 9 (60.0%)
		NEG: 0 (0.0%)	NEG: 9 (24.3%)	NEG: 47 (37.3%)	NEG: 21 (70.0%)	NEG: 6 (40.0%)
		*P* = .333	***P* < .001**	***P* < .001**	***P* = .002**	*P* = .273
	External	POS: 0 (NA)	POS: 13 (100.0%)	POS: 112 (58.0%)	POS: 2 (50.0%)	POS: 0 (NA)
		NEG: 0 (NA)	NEG: 0 (0.0%)	NEG: 81 (42.0%)	NEG: 2 (50.0%)	NEG: 0 (NA)
		*P* = NA	***P* < .001**	***P* < .001**	*P* = 1.000	*P* = NA
	Total	POS: 2 (33.3%)	POS: 59 (64.8%)	POS: 254 (60.5%)	POS: 55 (57.3%)	POS: 11 (64.7%)
		NEG: 4 (66.7%)	NEG: 32 (35.2%)	NEG: 166 (39.5%)	NEG: 41 (42.7%)	NEG: 6 (35.3%)
		*P* = .567	***P* < .001**	***P* < .001**	***P* = .043**	*P* = .086

For each emotion, the percentage is relative to the total number of positive or negative counts per emotion state (column-wise). In total, the percentage is relative to the total number of positive and negative counts. *P*-value is calculated as a difference in distribution between positive and negative counts using the Chi-square test. Bold values indicate significane (p < 0.05).

#### Correlation directionality and physiological dynamics

**Figure 6:**
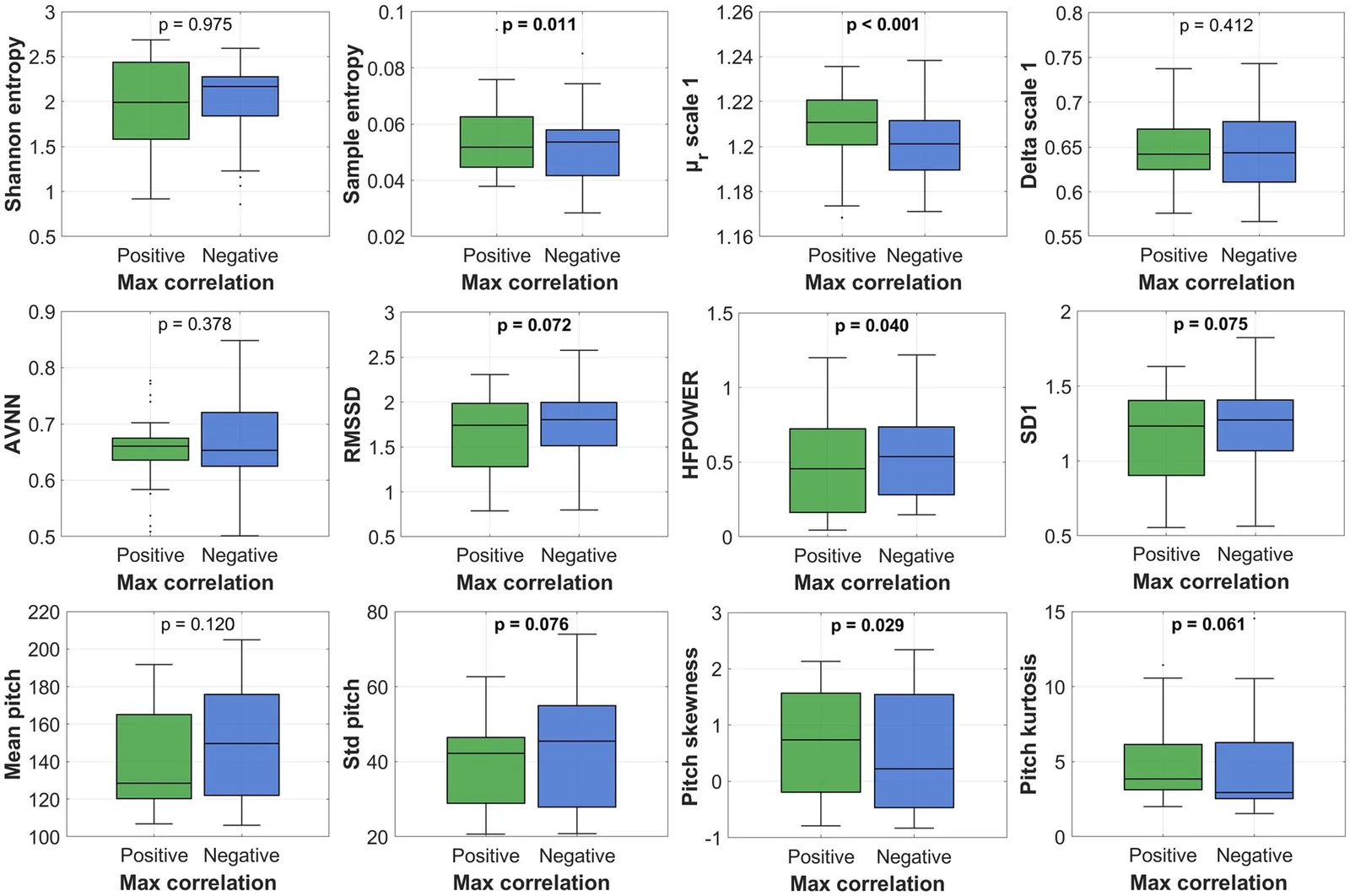
Significantly different features (based on lag) extracted from peak-to-peak (RR) intervals at positive and negative cross-correlation across all speaking-listening phases. Significance was tested through the Kruskal–Wallis test. Positive correlation indicates that both signals tend to vary in the same direction over time, whereas negative correlation indicates inverse coupling.

The strength of the cross-correlation also exhibited distinct patterns (Fig. 6 and Table 1). For example, HRV features followed a similar trend to that observed with lag, where negative segments generally showed higher amplitudes in RMSSD, HF Power, and SD1. However, the statistical differences were less pronounced. However, AVNN demonstrated higher median values with positive correlation (0.660 [0.636–0.675]). Regarding entropy analysis, Shannon entropy was higher for negative segments (2.168 [1.843–2.276]), and sample entropy was also elevated (0.054 [0.042–0.058], *P* = .011). Results for the diffusion analysis indicated a positive correlation with higher μ_r_ (1.211 [1.201–1.221], *P* < .001). Pitch characteristics followed trends similar to those observed with lag, where the mean and standard deviation of pitch were higher in negative segments. In contrast, skewness and kurtosis were higher in positive segments.

#### Correlation directionality and emotional states

**Figure 7:**
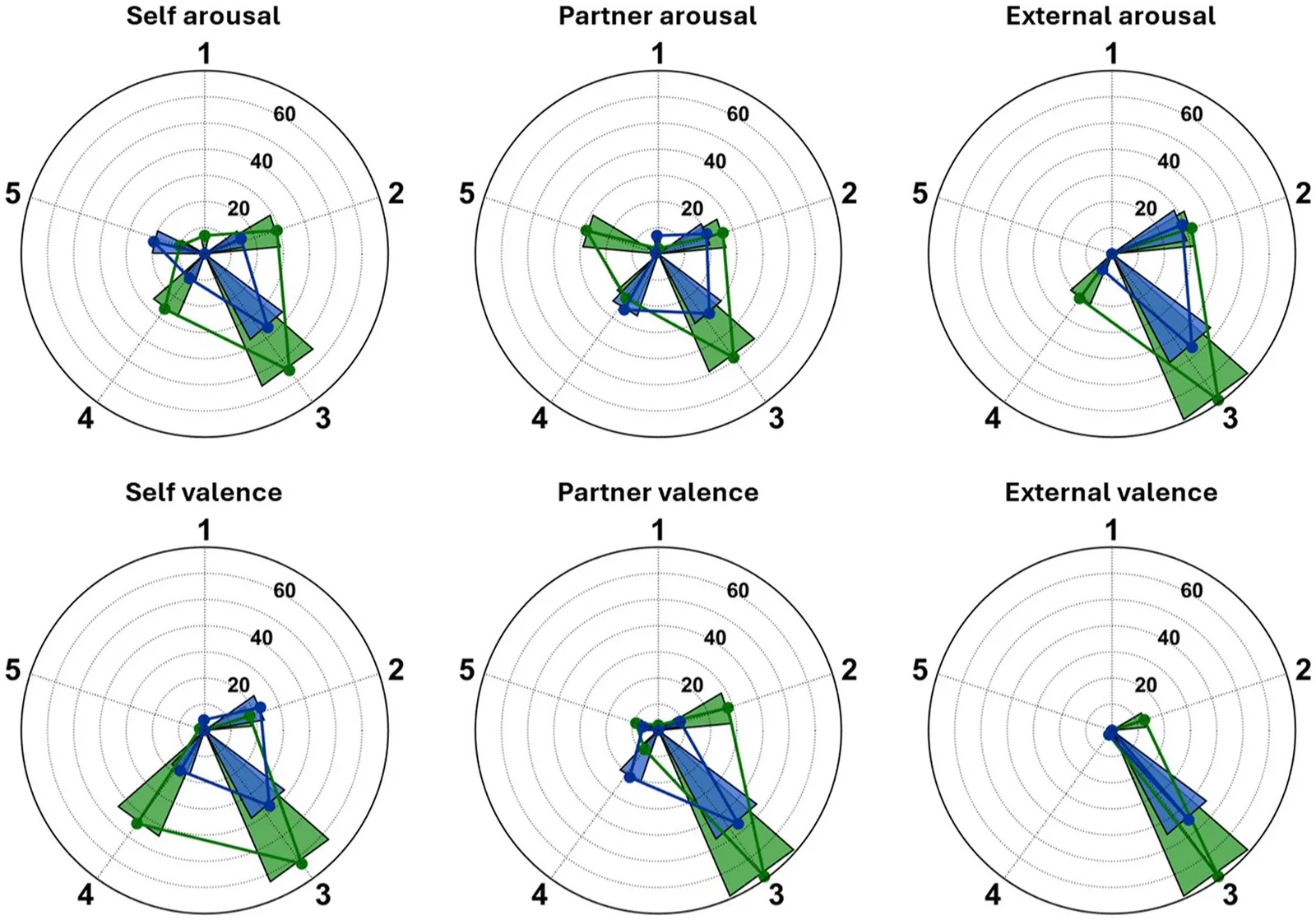
Distribution of positive and negative segments defined by correlation relative to emotional states in arousal and valence on a scale from 1 to 5. Segments were defined based on speaking-listening phases and matched with their corresponding dominant emotional state. Positive correlation indicates that both signals tend to vary in the same direction over time, whereas negative correlation indicates inverse coupling. Arousal reflects the level of emotional activation or intensity, ranging from low (1—calm) to high (5—excited), whereas valence represents the emotional polarity of the experience, ranging from negative (1—low values) to positive (5—high values).

Differences in emotional states relative to correlation directionality revealed more pronounced trends (Fig. 7 and Table 2). Self-reported arousal and valence showed a higher proportion of positive segments for states 3 and 4. However, the opposite trend was observed for lower emotional states, such as state 2, which exhibited more positive segments for arousal (*P* = .001) and more negative segments for valence (*P* = .029). As with lag directionality, the extreme emotional state (state 5) showed more negative segments in arousal (*P* = .010). State 3 followed a similar pattern for partner arousal and valence, with negative segments for arousal. For partner arousal and valence, state 3 followed a similar pattern, with more positive segments. However, in the extreme state (state 5), more positive segments were observed. Lower states, such as state 2, also displayed a higher proportion of positive segments (*P* < .001). External evaluations were more distributed than lag, with correlations showing stronger positive effects, especially for state 3 in terms of arousal and valence (*P* < .001). In general, positive lags became more prevalent as emotional intensity increased.

## Discussion

This study revealed the complex interplay among physiological, speech, and emotional systems during real-time social interactions. It highlights how emotions and physiological states are transmitted between speakers and how individual physiological, emotional, or speech traits can influence others. Participant connections as speakers and listeners, and measured by physiological, emotional, and speech characteristics play a role in decision-making and conversation outcomes. We highlight that leading or lagging roles in speaker–listener dynamics are linked to different physiological responses. Understanding conversational flow is important in various settings, such as debates, teacher–student interactions in classrooms, and therapy, as part of the multifaceted nature of interpersonal coordination.

Bidirectional lags were closely linked to ANS flexibility and complexity in contexts and social interactions. Negative lag segments indicated that the partner’s (often the listener) physiological changes preceded those of the first participant (or self, often the speaker) and were associated with increased parasympathetic activity, resulting in higher RMSSD and HF power. These segments were also associated with greater signal complexity, reflected in elevated Shannon entropy measures. This suggests that when a participant is responding to the partner’s leading influence, the physiological and emotional characteristics are not passively following. Instead, their physiological system is more flexible and adaptive, dynamically adjusting to the ongoing interaction. In contrast, positive lag segments, which reflect instances where the first participant leads the interaction, were characterized by distinct entropy profiles, e.g. higher sample and diffusion entropy, potentially indicating increased demands related to exerting control or driving the interaction. The elevated diffusion entropy suggests more complex, scale-dependent fluctuations in physiological dynamics, which may reflect the cognitive and regulatory effort required to guide and influence the partner’s responses over time. Additionally, because cognitive processes drive speech production, applying diffusion entropy analysis, we detected the key events associated with these deviations, and thereby distinguishing positive lag segments from negative ones.

Importantly, not all observed associations can be interpreted as interpersonal coupling in a strict mechanistic sense. Some effects likely reflect individual physiological or speech-related responses to the interaction context itself, such as cognitive load, speech production demands, or momentary emotional engagement, rather than direct bidirectional synchronization between interlocutors. In this study, coupling is considered as structured temporal alignment between participants’ signals, quantified through cross-correlation strength and directionality across speaking–listening phases. However, such alignment does not imply a single causal mechanism. Instead, coupling likely emerges from the interaction of multiple processes, including shared conversational demands, adaptive autonomic regulation, and affective responsiveness.

Emotional states were closely intertwined with patterns of directionality, highlighting the bidirectional relationship between affective dynamics and interpersonal coordination. Negative lag segments were more frequently observed during low emotional states (states 1 and 2), particularly those characterized by reduced arousal and engagement, suggesting that lower emotional activation may foster reactive or adaptive adjustments to the behavior of the debate partner. In contrast, positive lag segments were more often associated with moderately to high emotional states (states 3 and 4), indicating that exerting influence aligns with more balanced affective engagement between the debate dyad.

Our study has several limitations. First, while the integrated physiology-speech-emotion analysis successfully revealed distinct interaction characteristics, the sample size and context were relatively constrained, as the interactions were drawn from a structured debate-based database. This controlled setting may not fully capture the variability and complexity of naturalistic conversations. Second, although cross-correlation revealed significant associations, they do not establish causality. Future research employing experimental manipulations or causal modelling approaches will be necessary to clarify the direction of these influences. Future studies can address the findings presented in this study, derived from two complementary analytical methods focused primarily on dyadic interactions. This focus limits the generalizability of the results to larger groups or more dynamic, multi-person exchanges of physiological and emotional processes. Further validation of wider datasets is required in order to expand knowledge of conversational interactions across multiple systems. Third, the K-EmoCon dataset consists of a small and gender-imbalanced sample, which precluded reliable analysis of age- or gender-related effects. Consequently, the observed interaction patterns may partly reflect gender-specific dynamics and should be interpreted with caution. Future research should incorporate larger and more gender-balanced samples to enable systematic investigation of age- and gender-related effects. Finally, while we have noted significant findings related to speech pitch features, these effects may partly reflect shared speech production or conversational dynamics rather than purely bidirectional physiological or emotional coupling, underscoring the need for future multimodal analyses incorporating semantic information.

## Conclusions

In conclusion, this study demonstrates how physiological, speech, and emotional data dynamically interact during real-time social exchanges at different phases of speak-listen segments during a conversation, revealing distinct patterns of leadership, responsiveness, and coordination. By linking directionality and coupling strength to autonomic regulation, signal complexity, and emotional states, our findings highlight the multidimensional nature of interpersonal communication. These insights provide a foundation for future research and practical applications aimed at enhancing social interaction, from therapeutic settings to collaborative and educational environments.

## Data Availability

The data underlying this article (K-EmoCon) are available in Zenodo at https://zenodo.org/records/3762962.
